# Latent scurvy with tiredness and leg pain in alcoholics: An underestimated disease three case reports

**DOI:** 10.1097/MD.0000000000008861

**Published:** 2017-11-27

**Authors:** Christine Lux-Battistelli, Daniel Battistelli

**Affiliations:** Nouvelle Clinique des Trois Frontières, Saint-Louis, France.

**Keywords:** alcoholic patient, alcoholic withdrawal, ascorbic acid, leg pain, scurvy, vitamin C, weakness

## Abstract

**Rationale:**

Scurvy is often diagnosed at the state of well-established signs as, for example, skin and visceral purpura, gums involvement, loss of healthy teeth, which derive mostly from disturbance of collagen metabolism. Little is known about the state of latent scurvy, which symptoms are nonspecific and may mimic more common conditions such as weakness, leg pain, and muscle aching.

**Patient concerns:**

We report 3 cases of extreme lassitude and leg pain in alcoholics. In 2 of the 3 cases, discreet classic symptoms such as petechiae or hyperkeratosis of the legs involving collagen metabolism were also present.

**Diagnoses:**

Latent scurvy has been diagnosed thanks to historical experimental data reporting and undetectable ascorbic acid levels.

**Interventions:**

In addition to the treatments recommended by the French Alcohol Society, patients were given oral vitamin C 500 mg to 1000 mg per day for at least three months.

**Outcomes:**

Vitamin C supplementation allowed the regression of the symptoms, greatly improved the quality of life, and gave the possibility to return to work. Cartinine, requiring vitamin C for its hydroxylation, is an essential cofactor in the transport of long-chain fatty acid into mitochondrial matrix. Therefore, it plays an important role in energy production via beta-oxidation. It is thought that carnitine metabolism impairment is responsible for weakness or muscle aching.

**Lessons:**

We recommend being aware of the possibility of latent scurvy in chronic alcohol abusers. The vitamin C supplementation and dietetic recommendation eating fresh fruit and vegetables may help to cure tiredness and to return more easily to socialization and to work.

## 1 Introduction

Cases of scurvy are occasionally reported within the alcoholic population when clinical and well-established signs are observed, as skin and visceral purpura, gums involvement, loss of healthy teeth, which derive from disturbance of collagen metabolism.^[1,2]^ Nevertheless, there are nonspecific symptoms that can mimic more common conditions, such as lassitude, weakness, irritability, and scurvy is often underestimated.^[3,4]^

We report 3 cases of extreme weakness and pain of the lower legs, in addition to petechial purpura of the lower legs in one case and edema in another case, induced by scurvy in alcoholics.

## 2 Case reports

### 2.1 Case report 1

A 54-year-old man presented in dermatology for psoriasis present for years, but his major complain was weakness and pain in the right lower leg, which appeared when he stood a long time, disappearing when he was lying down. This tiredness and pain prevented him from going back to work and he was afraid to loose his position as an electrician.

He was hospitalized 3 months ago for gastric pain, extreme weakness, and icterus. He did not take any medication before. He started to drink a few years ago as a result of depressive reaction after the loss of his previous job. Routine observation on admission noted a heart rate of 110/minute with normal temperature, abdominal and cardiovascular examination that were normal, and no neurologic deficiencies. Psoriasis was diffuse involving the nails.

Laboratory findings on admission revealed an acute hepatitis [aspartate aminotransferase (AST) 16,658 IU/L (reference range <50), alanine aminotransferase (ALT) 1055 IU/L, (reference range <50) with conjugated bilirubin at 20 mg/L (reference range <3) in correlation with the abdominal pain and the icterus, alcoholhemia (1.60 g/L)]. The other primary investigations were in normal range and no other cause of hepatitis than alcohol was found. Abdominal ultrasound examination was normal. Madrey score was 8.41, so no general corticotherapy was introduced. Two days after alcohol withdrawal, hepatitis improved quickly (AST/ALT 3857/769). Four days later, hepatitis continued to improve (AST/ALT 176/273). The patient was discharged 3 weeks later with no more hepatitis. His treatment was vitamin B1 therapy 750 mg/day, naltrexone 50 mg/day, oxazepam 20 mg/day during 1 week more and enalipril-lercanidipine for hypertension.

Two months later after hospitalization, tiredness was still present with pain in the right leg that disappeared when lying down, without any objective sign of neuropathy. His deep tendon reflexes were intact. Petechial purpura appeared one moment briefly on the legs, the lower legs were scaly, and gums were swollen.

Due to the apparition of purpura without any thrombocytopenia or any coagulation disorder, diagnosis of scurvy was suspected. After the informed consent of the patient was obtained, ascorbic acid serum level was measured. It was significantly decreased at 3.1 μmol/L (normal range 26.1–84.6) despite usual nutrition since the hospitalization.

Vitamin C supplementation at 1000 mg/day cleared the purpura in 1 week. But 8 to 10 weeks were necessary to cure the weakness and the pain when standing a longer time. The patient returned to work at this moment.

### 2.2 Case report 2

A 24-year-old man was hospitalized for functional disability consecutive to edemas of lower limbs. He had an ancient history of drug addiction 5 years ago, and was actually clean with replacement therapy by buprenorphine. He had also a history of alcohol intoxication, which he tried to stop 1 year ago during hospitalization. Despite the medication of acamprosate, conventional treatment of high-dose of vitamin B1, and psychological follow-up, he started drinking again with an alcohol intake of 2 to 4 L of wine per day and sometimes vodka, but he stopped by himself 4 days before the hospitalization.

On physical examination, he had edemas of the lower limbs with a body-weight gain of 10 kilos during the previous year. He could only move with difficulty and with the help of crutches with a dyspnea in the slightest effort. During the hospitalization, he was very tired, moving from his chair to bed and from his bed to his chair but he was oriented, with no diplopia, no short attention span, no reduced speech spontaneity, confusion, or drowsiness. He also complained of dysesthesia of the 4 legs, especially on the extremities. He had no petechial or purpuric rash. Cardiovascular examination was normal.

Laboratory analyses revealed that red and white blood cells count, creatinine clearance, serum level of albumin and pre- albumin, liver function, were within normal ranges, as was the 24 hours proteinuria. The following were slightly disturbed: sodium at 132 mmol/L, gamma glutamyl transferase (γGT) 103 UI/L, and mean corpuscular volume (MCV) at 111 μm^3^.

Electromyography did show a symmetric, mixed axonal sensorimotor polyneuropathy in the lower limbs, and axonal sensory neuropathy of the hands.

As during the previous hospitalization, he did receive vitamin B1 therapy at the dose of 750 mg/day per os and diuretics, the last allowing partially edemas resorption with a little effect on the tiredness (he managed to walk 100 m without crutches).

After a sobriety period of 4 months, he started drinking again and was readmitted to hospital for tiredness, minima edemas, difficulty of walking, aching of muscles of the legs, and dysesthesia of both legs and hands.

Laboratory findings revealed albumin, pre-albumin, and renal function within normal ranges, a hepatic cytolysis twice the normal range, and γGT 5 times the normal range. He was given as recommended vitamin B1 (750 mg /day per oral) and 150 mg of oxazepam per day by the guidelines in France. Vitamin B1, collected 1 week after the admission, was 376 nmol/L (reference range 66–220), and vitamin B6 collected at the same time was 41 nmol/L (reference range 15–73). The similarities of pain and tiredness, even worse, with the first patient made us suspect scurvy. After patient's informed consent, vitamin C also collected 1 week after admission was <3 μmol/L (undetectable levels on vitamin C). He was given oral vitamin C 1000 mg per day. The tiredness diminished 3 days after the beginning of the treatment and he was discharged 1 week after because he drunk again vodka in his room. However, he got recommended to take the vitamins and fresh fruits each day.

Long-term follow-up showed a great improvement of his tiredness without any difficulties for walking. Dysesthesia still needed pregabaline for sometime.

### 2.3 Case report 3

A 48-year-old man presented in dermatology for prurigo that had begun 1 year ago, treated by local corticosteroids, as well as hyperkeratosis of the lower limbs.

He had a long history of alcohol intoxication with 1 bottle of whisky daily during the last years. He has been hospitalized 4 months ago for alcohol withdrawal with success. He also had a myocardial infarction 5 years ago with the revealing of diabetes and a 30 pack-year smoking history. His treatment was naltrexone (for the management of alcohol dependence), clopidogrel, rosuvastatine, ramipril, atenolol, sitagliptine, glimepiride, and insulin.

The patient also complained being unsteady on his feet, with the necessity of lying down on the sofa after 3 hours. He recalled teeth's loss of the upper jaw 3 years ago, and recently, on the lower jaw, teeth bare from the looseness of the gums, needing dentures.

**Figure 1:**
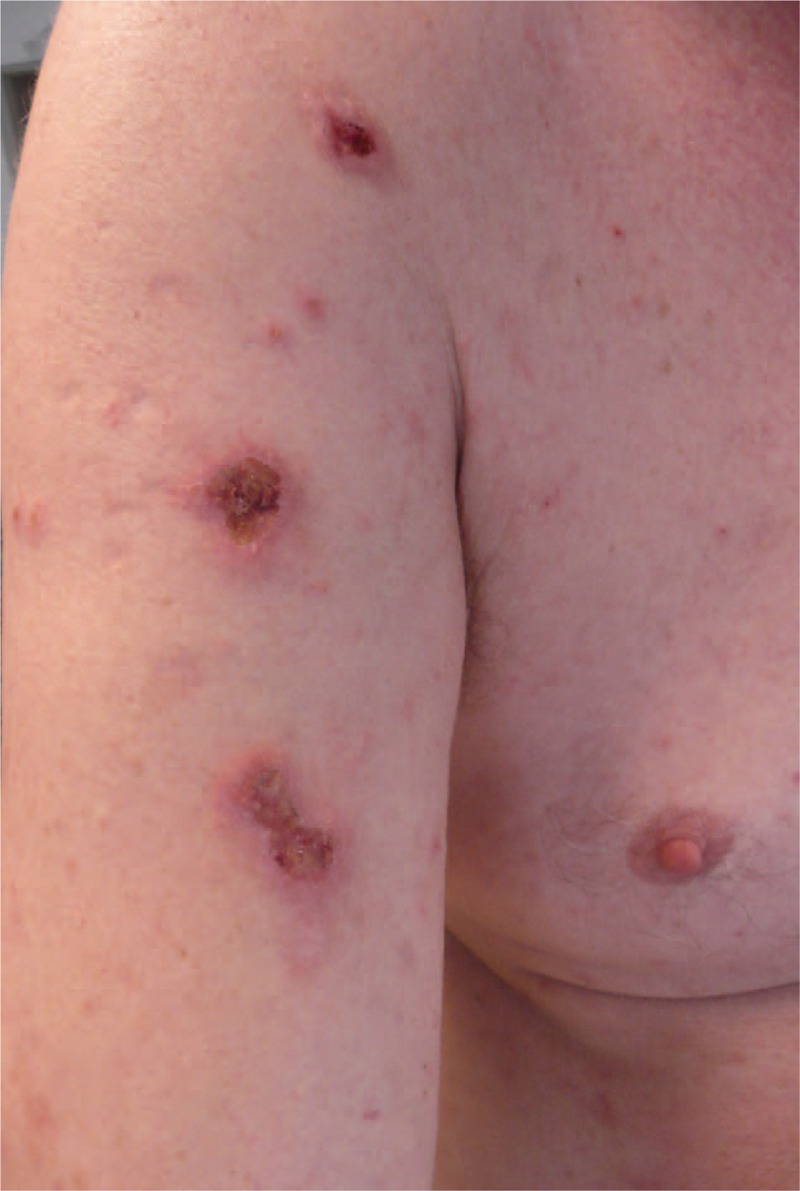
Case report no. 3; Large lesions of prurigo due to intercurrent scabiose.

**Figure 2:**
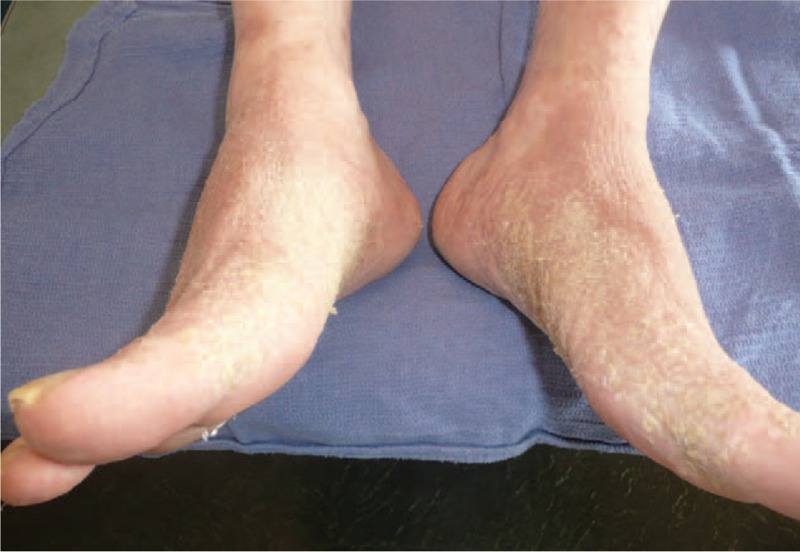
Case report no. 3; Hyperkeratosis of the inner part of the feet. Follicular keratosis can be seen on the shin.

On physical examination, large lesions of prurigo were observed on the upper part of the body (Fig. 1). Hyperkeratosis of the legs, especially on the inner part of the feet, and follicular hyperkeratosis on the lower limbs were also observed (Fig. 2).

After the patient's informed consent, it was decided to measure vitamin C and to begin vitamin C supplementation even before knowing the result (results are obtained in approximately 10 days in France). Therefore, the patient was eager to begin the treatment and the serum was unfortunately collected 3 days after beginning of vitamin C therapy (ascorbic acid 500 mg per day). Vitamin C was slightly under normal range at 24.1 μmol/L, and vitamin B1 was within normal range at 165.3 nmol/L (reference range 66.5–200) as vitamin B6 at 29 nmol/L (reference range 15–73).

The hyperkeratosis of the lower legs was cleared 3 weeks after the introduction of vitamin C therapy, the tiredness was still present, but the patient volunteered to go again to work. One month later, after beginning of PUVA-therapy (Psoralen+UVA-therapy) to reduce the itching, it appeared that the cause of the prurigo was scabies with the emergence of 2 acarine burrows that were not excoriated and 1 acarine burrow in his daughter. Scabies were treated, but PUVA-therapy needed to be continued to cure entirely the prurigo.

One month later, he went back to work but still with tiredness at the end of the day. Burning sensation of the feet disappeared at the same time. Tiredness in the evening relapsed 2 months after the cessation of vitamin C therapy and disappeared 2 weeks after its reintroduction.

## 3 Discussion

Scurvy is an old disease that have given a lot of precise description by ancient physicians especially from the north of Europe, that is, Bachstrom, Boerhaven, Van Swieten, Lind, and so on. It was Lind's merit to have demonstrated that lemon was the most active among other medication to cure scurvy in young sailors.^[5]^ Boerhaven in its aphorisms has described the course of the disease in 4 steps from the beginning to the end, but he added that the symptoms did not appear exactly on the same order in each patient.^[6]^The description of the first step was “ An unusual laziness, stiffness, an inclination to sit and lie down, a spontaneous weariness all over, a general heaviness, a pain of the muscles as after to great a fatigue, chiefly in the legs and loins, a great difficulty in walking chiefly up or down stairs, and in the morning upon first waking in all the limbs and muscle a sense of weariness and a smarting.”^[6]^

The description of the second step was the “ difficult, panting and almost deficient breathing of upon every little motion, the swelling of the legs coming and going,” “the gums painful, the teeth bare from the retraction and looseness of the gums, and the red, yellow and purple spots.”^[6]^ The last symptoms are the description of most of the cases of scurvy presently observed.^[1,2,7]^

The third step was described as “ the gangrene, ulcers and looses of the teeth…,” and the 4 steps as “the black spots, vomiting of blood and voiding the same in great quantities by stool, a putrefaction.”^[6]^

Regarding Hodges et al,^[8]^ in “ Experimental scurvy on man,” they studied the first step or latent scurvy among 6 volunteering prisoners in good health during the year 1966. Two of them escaped the 54th day of deficiency, but on the 4 remainders, the same symptoms of vitamin C deficiency appeared before the onset of skin purpura. This was tiredness, especially of the lower limbs, and mild general malaise that begun insidiously. It was present in all 4 subjects and necessitated a reduction of their 10 miles daily walking. Two of them complained of dull aching muscular pain in both legs. One of them also complained of muscular aching in the shoulder region. The observers added that prisoners are inclined to exaggerate any discomfort, but these signs were judged to be worthy, as they appeared about the time of development of the first objective manifestations of scurvy, namely hyperkeratosis, petechial hemorrhages, and congestion of follicles. It is of note that, as ancient physicians described it, the same manifestations did not appear in all patients, but only part of them did have hyperkeratosis, a few other petechial hemorrhages, or gum swelling, or bleeding gums.

Fatigue was also the first symptom to appear according to Crandon et al,^[9]^ who experienced scurvy on himself. To avoid interferences with other lack of vitamins, he took vitamin supplement except vitamin C. Weakness was first noticed about the end of the third month of vitamin C privation, and become progressive as time went on. It was measured objectively by a run test that was severely disrupted during the vitamin C privation: he was only able to run during 16 seconds, whereas following ascorbic acid therapy during 10 days at the dose of 1000 mg intravenously, he ran for 66 seconds. This performance was much lower than the 270-second average of men of his age. Seven weeks after vitamin C reintroduction, this performance was still lower at 84 seconds. Concerning moderate work, the subject could perform duration of 4.5 minutes during the scorbutic state, and the same duration 10 days later and 7 weeks later; meanwhile, the average duration of the same work for men of his age was 15 minutes.^[9]^

Lower extremity pain was also the major reason of difficulty walking by 3 children.^[10]^ The same symptoms are described among 6 adults, unable to walk unaided because of the pain in their legs. Some of them had also “woody” edema of the lower legs and some scaling of the same area. Most patients did have in addition more characteristic features of scurvy (purpura, bruises), but 1 patient with edema and scaling and marked limitation of the left knee did not have any visible hemorrhages into the skin.^[11]^ Furthermore, 3 of these 6 cases had the classic follicular hyperkeratosis on the limbs, and 3 presented scaling involving the lower legs or the shin and dorsum of the feet.

These descriptions of the first manifestations of scurvy are very similar with the symptoms our 3 patients complained about. To support the diagnosis of scurvy, one of them (patient case report 1) had petechiaes that faded quickly with vitamin C therapy and the other one (patient case report 3) scaling on the lower legs that disappeared after 3 weeks of vitamin C supplementation. This patient lost previously all his teeth, so gum involvement could not appear.^[12,13]^

It might seem surprising that 2 to 3 months were needed to cure the tiredness when only 1 month, sometimes less, was needed to the disappearance of the purpura.^[1,14]^ Crandon et al,^[9]^ on his self-experimentation of scurvy, did particularly well describe that little bleeding manifestations as petechiae on the leg did fade after 1 week of intravenous vitamin C therapy, and that, on the contrary, normalizing the run test needed more than 7 weeks. Also, in childhood scurvy, skin lesions, gum swelling, and ambulating improved in 1 month, but it needed 3 months to come walking to the clinic.^[14]^

Ascorbic acid is required as a cofactor for hydroxylation of procollagen molecules. The absence of hydroxypropyl and hydroxylysyl residues renders the nascent polypeptide instable. This abnormality affects blood vessel integrity because of impaired synthesis of basal laminae, media, adventice, and surrounding connective tissues. The consequences are perivascular edema, protrusion of endothelial cells into the vascular, and erythrocyte extravasation.^[13]^ This leads to the well-known hemorrhagic manifestations such as follicular hemorrhages, purpura, and partially to edema to the lower limbs.

Vitamin C is also essential for the metabolism of tyrosine, the synthesis of peptides hormones, norepinephrine, and, what is of interest for us here, carnitine. Carnitine, requiring vitamin C for its hydroxylation, is an essential cofactor in the transport of long-chain fatty acid into mitochondrial matrix. Therefore, it plays an important role in energy production via beta-oxidation. It has been suggested that carnitine deficiency is responsible for the early symptoms of scurvy such as lack of energy and muscle aching.^[15,16]^

The hard swelling ascribed to scurvy in our 24-year-old patient (Case report 2) might have arisen from combined thiamin deficiency causing cardiac disease. But this patient did not have any signs of cardiac insufficiency (no hepatomegaly, no hepatojugular reflux, normal cardiac examination). Moreover, at each hospitalization, he took high-dose vitamin B supplementation as recommended by the French guidelines.^[17]^ Leg pain and edema occurred among patients in chronic scurvy.^[11]^ The edema was also observed in experimental scurvy among the Iowa prisoners, by which there was no vitamin B1 deficiency. Hard swelling over the left thigh with leg pain was also described in a child who refused to walk with scurvy as the only cause to his symptoms.^[14]^

It is not our intention to deny the role of a chronic vitamin B1 deficiency. In our 3 patients, combined deficiencies of vitamin B1 and vitamin C appear as the most likely hypothesis with the intricacy of the symptoms: the burning sensation of the feet is more relevant for vitamin B1 deficiency confirmed by electromyography.^[18]^ These dysesthesias are frequently accompanied by dull pain and cramps in both feet and calves.^[18]^ But leg pain can also be connected to vitamin C deficiency and the more characteristic of latent scurvy will be the profound lassitude as described also by Veerapaneni et al.^[19]^

Vitamin B1 deficiency risk is well known in chronic alcohol abusers. Vitamin B1 is systematically given during hospitalization in France and in the UK,^[7]^ whereas vitamin C deficiency risk does not require systematic attention. Vitamin C supplementation will be given when symptoms of evident scurvy are present, such as purpura or bleeding gums. But symptoms of latent scurvy such as profound tiredness or leg pain can mimic more common diseases, being entangled in vitamin B1 deficiencies or ascribed to psychotropic overdose and so can be neglected.

We recommend being aware of the possibility of latent scurvy in chronic alcohol abusers. The vitamin C supplementation and dietetic recommendation eating fresh fruit and vegetables may help to cure tiredness and to return more easily to work.

## 4 Conclusion

Our 3 case reports highlight the difficulties to diagnose latent scurvy at a stage where the typical clinical manifestations of scurvy are not present. Nevertheless, these clinical symptoms such as profound tiredness, difficulties to walk or to stand for a long time, pain in the lower legs, may deeply impact the quality of life of patients who succeeded in alcohol withdrawal.

Recognition of these symptoms may allow the clinical diagnosis, supported by the determination of ascorbic acid level. Vitamin C supplementation will allow the improvement or resolution of the symptoms and thus to consolidate the diagnosis of latent scurvy. Moreover, social reinsertion of the alcohol withdrawal patient will be facilitated.
